# Online gaming dependency, attention levels and sleep quality among online gamers

**DOI:** 10.1192/j.eurpsy.2021.1530

**Published:** 2021-08-13

**Authors:** G. Esgalhado, A. Fernandes, H. Pereira

**Affiliations:** 1 Psychology And Education, University of Beira Interior, Covilha, Portugal; 2 Psychology And Education, University of Beira Interior, covilha, Portugal

**Keywords:** Online gaming dependency, Level of attention, Quality of sleep

## Abstract

**Introduction:**

The persistent and recurrent use of the internet to engage in games, often with other players, which leads to clinically considerable deficits and online dependency still lacks more research support its impact of attentional levels and Sleep Quality.

**Objectives:**

To assess levels of Online Gaming Dependency, its impact on attention levels and quality of sleep among online gamers.

**Methods:**

The following instruments were used: a sociodemographic questionnaire, as well as the Video Game Behavior Assessment Scale (α = .842) and the Portuguese version of Color and Words Stroop Test. The sample consists of 66 individuals, 92.3% male and 7.7% female, with an average age of 23.1 (SD ± 4.07).

**Results:**

Mild levels of online gaming dependency were obtained. Male gamers presented higher motivation to play online games, and higher levels of Stroop interference. Regarding the association between gaming behaviors and quality of sleep, results show that individuals who have difficulty falling asleep show greater motivation to play online games, a greater degree of concern with daily life and a greater frequency of negative behaviors in relation to online gambling.

**Conclusions:**

The results show the importance of an increase in investigations in this area, as well as to the development of psychological assessment instruments and psychological intervention strategies, based on scientific evidence, on the different variables under study, in order to improve the quality of life in of individuals.

